# Modulating Chemosensitivity of Tumors to Platinum-Based Antitumor Drugs by Transcriptional Regulation of Copper Homeostasis

**DOI:** 10.3390/ijms19051486

**Published:** 2018-05-16

**Authors:** Yu-Hsuan Lai, Chin Kuo, Macus Tien Kuo, Helen H. W. Chen

**Affiliations:** 1 Department of Radiation Oncology, National Cheng Kung University Hospital, College of Medicine, National Cheng Kung University, Tainan 70428, Taiwan; coscoscos.tw@hotmail.com (Y.-H.L.); tiffa663@gmail.com (C.K.); 2 Institute of Clinical Medicine, College of Medicine, National Cheng Kung University, Tainan 70428, Taiwan; 3 Department of Translational Molecular Pathology, The University of Texas MD Anderson Cancer Center, Houston, TX 77054, USA; tienkuo@sbcglobal.net; 4 Center of Applied Nanomedicine, National Cheng Kung University, Tainan 70101, Taiwan

**Keywords:** high-affinity copper transporter, *hCtr1*, Sp1, cisplatin, ovarian cancers, drug-resistance

## Abstract

Platinum (Pt)-based antitumor agents have been effective in treating many human malignancies. Drug importing, intracellular shuffling, and exporting—carried out by the high-affinity copper (Cu) transporter (*hCtr1*), Cu chaperone (Ato x1), and Cu exporters (ATP7A and ATP7B), respectively—cumulatively contribute to the chemosensitivity of Pt drugs including cisplatin and carboplatin, but not oxaliplatin. This entire system can also handle Pt drugs via interactions between Pt and the thiol-containing amino acid residues in these proteins; the interactions are strongly influenced by cellular redox regulators such as glutathione. *hCtr1* expression is induced by acute Cu deprivation, and the induction is regulated by the transcription factor specific protein 1 (Sp1) which by itself is also regulated by Cu concentration variations. Copper displaces zinc (Zn) coordination at the zinc finger (ZF) domains of Sp1 and inactivates its DNA binding, whereas Cu deprivation enhances Sp1-DNA interactions and increases Sp1 expression, which in turn upregulates *hCtr1*. Because of the shared transport system, chemosensitivity of Pt drugs can be modulated by targeting Cu transporters. A Cu-lowering agent (trientine) in combination with a Pt drug (carboplatin) has been used in clinical studies for overcoming Pt-resistance. Future research should aim at further developing effective Pt drug retention strategies for improving the treatment efficacy.

## 1. Introduction

**Figure 1:**
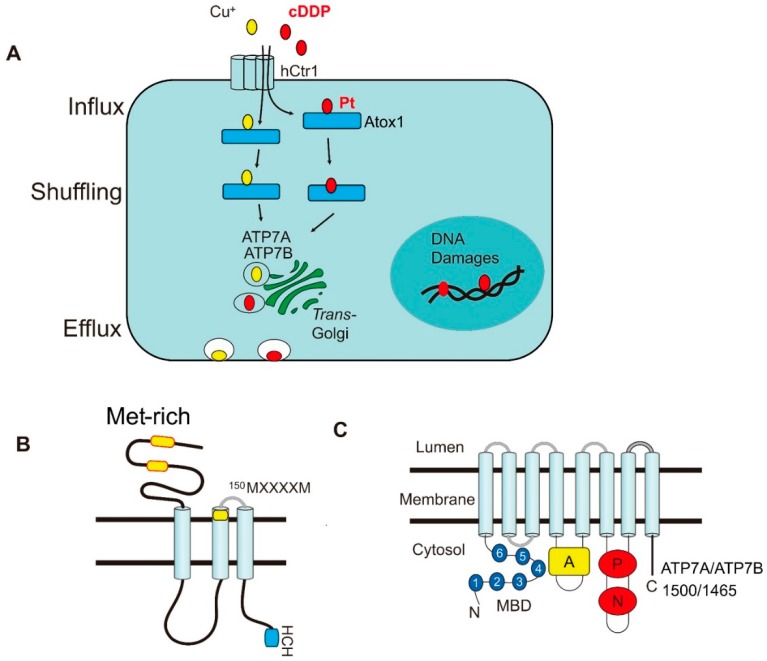
The similarity of transport systems between Cu(I) and cDDP. (**A**) routings of Cu(I) and cDDP from influx, intracellular trafficking (shuffling), to efflux; (**B**) schematic diagram depicting structure of *hCtr1*; (**C**) structure of ATP7A and ATP7B.

Platinum (Pt)-based drugs represent an extraordinary accomplishment in antitumor inorganic metal drug development [1]. They have been the mainstay of cancer chemotherapy in many different types of human malignancies for the last four decades since FDA approved *cis*-diamminedichloroplatinum(II) (cisplatin, cDDP) in 1978 [1,2]. These drugs are effective in treating advanced testicular cancers and ovarian cancers with cure rates of about 90% and 70%, respectively [3]. Together with carboplatin (Cbp) and oxaliplatin (Oxl), these Pt-based agents have shown a wide spectrum of antitumor activities including cancers of lung, bladder, breast, colon, and head and neck. It has been recognized that Pt drugs can attack many cellular targets including the plasma membrane, cellular organelles (mitochondria and endo-lysosomes), endoplasmic reticulum and cytoskeleton (see review in ref. [4] and references therein), but DNA damages are the principle cause of Pt drug-induced cell lethality. Another important factor that affects the treatment efficacy is the transport system which includes drug accumulation, intracellular drug shuffling and drug efflux (Figure 1A). This system cumulatively regulates the steady-state of intracellular drug contents which are directly correlated with the extent of DNA damage and cellular lethality [5,6]. Reduced cellular Pt content is an important hallmark of cDDP resistance in a wide variety of drug-resistant cell lines and tumors derived from Pt-refractory patients [7,8,9].

Previous works suggested that cDDP enters cells via a simple passive diffusion mechanism, because drug import is unsaturable and cannot be competed by cDDP analogues [6,10,11]. These findings suggest that cDDP influx may not require a transporter or carrier. However, because cDDP is a highly polar compound and its ability to across cellular membrane is limited, it is believed that the primary mechanism of Pt drug transport requires transporters. Indeed, studies have demonstrated that several copper (Cu) transporters are actively involved in the import, intracellular distribution, and export of Pt drugs [3]. These observations demonstrated that Cu ions and cDDP share similar transport mechanisms and mutually interfere with their cellular accumulations. In this review, we will first discuss the underlying mechanisms of how the Cu transporter system impedes Pt drugs from entering, following intracellular trafficking, and then exiting the cells. We will then focus on the transcriptional regulation of Cu transporter that bears clinical relevance to the treatment efficacy of Pt-based cancer chemotherapy.

## 2. Roles of the Copper Transporters in Pt Drug Transport Mechanism

### 2.1. Cisplatin Importers 

It has been established that the high-affinity copper transporter 1 (Ctr1, also known as SLC31A1) is the primary transporter for cDDP [3,12]. Ctr1 also transports Cbp with reduced efficacy, but does not transport Oxl. cDDP-resistant cell lines show reduced accumulation of cDDP but not Oxl or satraplatin (JM216), and no cross-resistance to these analogues [13]. The major influx transporters of Oxl are organic cation/carnitine transporter (OCT1 and OCT2) [14,15]. Ctr1 is an evolutionarily conserved Cu(I) ion transporter shared from yeast to humans. The important roles of Ctr1 in cDDP uptake were initially demonstrated by genetic ablations in yeast cells and in mouse embryonic fibroblasts, which exhibited decreased cDDP uptake and increased cDDP resistance [16]. This was also confirmed by the finding that overexpression of human Ctr1 (*hCtr1*) in small cell lung cancer cells increases cDDP uptake [17]. However, these observations may depend on cell sources, because overexpressing ectopic *hCtr1* was seen in cDDP-resistant cells but not in its drug sensitive cells [18,19]. While the precise mechanism of this discrepancy is not known, it may be relevant to the cellular capacity of Cu homeostasis and regulation mechanisms, which will be discussed below.

The *hCtr1* is a 190-amino acid membrane protein that contains three transmembrane domains with the N-terminus extracellularly located and the C-terminus inside the cytoplasm. Site-directed mutagenic analyses have identified multiple conserved amino acids in *hCtr1* that are critical for transport of Cu(I) and cDDP transports. These include ^40^MMMMxM in the N-terminal extracellular domain (where M denotes methionine and x denotes variable amino acids), ^150^MxxxxM in the second transmembrane domains [20], ^189^HCH in the cytoplasmic C-terminal domain, and others [21] (Figure 1B). However, mutations at the GG-4 motif (^167^GxxxG) reduces Cu(I) but not cDDP transport [20], suggesting the subtle differences in *hCtr1*-mediated transport between these substrates. It is suggested that cDDP (chemical formula: *cis*[Pt(Cl_2_(NH_3_)_2_)] interacts with the extracellularly exposed methionine (M)-motifs by forming the [Pt(Met)Cl(NH_3_)_2_] intermediate, resulting in induced *hCtr1* conformational changes and stabilization of the trimeric formation as revealed by protein cross-linking agents [20]. cDDP and Cu(I) are thought to transverse through the axis of the trimeric *hCtr1* channel and move inward by an intermolecular sulfur-sulfur exchange mechanism [3]. Electron cryostallography of 2D protein crystals in a native phospholipid bilayer provides an estimate of ~9 angstroms for the pore size of the *hCtr1* homotrimer configuration [22]. cDDP has two each of chloride (Cl) and aminonia (NH_3_) ligands coordinated to the central Pt in a square-planar structure. While the precise molecular dimension of cDDP is unclear, it is estimated to be at least three to four orders of magnitude (~1 nm) larger than the pore size [23], suggesting that conformational changes are involved in *hCtr1*-mediated cDDP passing. 

Humans also have a low-affinity Cu transporter hCtr2 (SLC31A2) arisen by gene duplication from *hCtr1* [24]. hCtr2 shares substantial structural homology with *hCtr1*, but has only 143 amino acid residues because the majority of the N-terminal Met-rich sequence is missing. *mCtr2*-knockout mice show elevated Cu accumulation in several tissues with increased mCtr1 expression [25]. Expression of hCtr2 is mainly localized in intracellular vesicles. Recent studies demonstrated that mCtr2 interacts with mCtr1 and causes truncation of mCtr1 through cleavage of its ectodomain by a cathepsin protease, resulting in substantially reduced Cu(I) and cDDP transport capacities, and thus affecting the effectiveness of cDDP killing [26]. However, another recent study using CRISPR-Cas9 genomic editing strategy to knockout hCtr2 in two human tumor cell lines demonstrated only modest changes in cDDP sensitivity compared to the parental cell lines [27]. The discrepancies of these results are yet to be determined. In 40 ovarian cancers, it was reported that while high *hCtr1* expression is associated with chemosensitivity of Pt-based drugs, patients with low *hCtr1* and high hCtr2 in their tumors have poor treatment outcomes and shorter overall survival (OS) time [28]. In another study, it also reported that ovarian cancer patients with high hCtr2/*hCtr1* ratios in the tumor lesions are resistant to Pt-based chemotherapy [29]. These results demonstrated that hCtr2, in conjunction with *hCtr1*, may also involve in chemosensitivity of Pt drugs.

### 2.2. Cisplatin Chaperone

Once inside the cells, cDDP, like Cu(I), is carried away by different Cu chaperones for intracellular delivery to various compartments [30]. Antioxidant protein 1 (Atox1), one of the Cu chaperones, receives Cu(I) directly from the C-terminal end of *hCtr1* at the cytoplasmic membrane. The Cu-Atxo1 complex delivers Cu(I) to the Cu-efflux pumps, ATP7A and ATP7B, which are two P-type ATPases and located at trans-Golgi network (TGN) [31,32,33] (Figure 1A). The driving force for this directional trafficking is thought to be due to affinity gradients between protein partners along the route [34].

The human Atox1 contains 68 amino acids and has a β_1_α_1_β_2_β_3_α_2_β_4_ ferredox-like structure. The Cu binding motif ^12^CXXC of Atox1 is located between the β_1_ ανδ α_1_ loops [35]. It has been demonstrated that cDDP also binds this motif when delivering cDDP to ATP7A/ATP7B efflux pumps [36]. Deletion of Atox1 results in resistance to cDDP, indicating its important role in Pt drug sensitivity [37,38]. 

Atox1 also contains a nuclear targeting signal (^38^KKTGK) between the α_2_ ανδ β_4_ loop for Cu-dependent nuclear translocation of Atox1 [39]. Nuclear Atox1 functions as a transcriptional regulator for the mammalian cell proliferation gene *cyclin D1* by interacting with the 5′-GAAAGA sequence about 500 bp upstream of the transcription start site. Other Atox1-regulated genes include extracellular superoxide dismutase (*SOD3*) which encodes a secretory Cu-containing antioxidant enzyme [40], and *OCT4* which codes for a pluripotency factor in embryonic development [41]. The nuclear targeting property of Atox1 may deliver cDDP to elicit its lethal effect of DNA damage.

### 2.3. Role of ATP7A and ATP7B in cDDP Efflux

ATP7A and ATP7B, contain 1500 and 1465 amino acids, respectively, each have 8 transmembrane domains (TMDs). They share 67% amino acid sequence identity. Both ATPases contain several functionally conserved domains, i.e., six metal-binding domains (N-MBD) at the N-terminus, each with a CXXC motif; the nucleotide-binding domain (N-domain) for ATPase catalytic activity; the P-domain for phosphorylation at the ^1207^Asp residue; and the A-domain for actuator/dephosphorylation (Figure 1C). There are also multiple Cu-binding sites located at transmembrane domain (TM) 4, TM5, and TM6 [42,43]. ATP7A is mainly expressed in the intestinal epithelium for Cu absorption from food. Mutations in ATP7A result in systemic Cu deficiency that causes the Menkes’ disease. ATP7B is mainly located in the liver and brain. Mutations of ATP7B result in massive Cu buildup in these organs, resulting in Wilson’s disease [44,45].

Detailed mechanisms of how Cu/Pt-Atox1 transfers the metal ions into ATP7A and ATP7B and how these metals are subsequently eliminated remain largely unknown. Current understanding suggests that similar protein folding of the CXXC metal binding motifs between Atox1 and the N-MBD of ATPases may facilitate rapid intermolecular metal transfer through electrostatics, hydrogen bonding, and hydrophobic interactions [46]. Another critical amino acid residue in Atox1 is ^60^lysine which is essential for protein heterodimerization with ATPases for processing Cu(I) transfer [47,48].

While Cu-Atox1 can potentially interact with all the six N-MBDs of ATP7A and ATP7B, it preferentially interacts with the N-MBDs 1 to 4 [42]. Interactions of Cu-Atox1 with these N-MBDs induce conformational changes and activate ATPase catalytic activity by mobilizing N-MBD to cross-talk with the N-domain of ATP7B [49]. These interactions induce autophosphorylation of Asp^1027^ in the P domain by ATP, and phosphorylation of several serine residues in the TMDs by protein kinase D (PKD). However, it was found that deletion of the first five N-MBDs did not suppress the autophosphorylation of Asp^1027^, whereas PKD-induced phosphorylation of the serine residues was downregulated [42,50]. Furthermore, recent results showed that native ATP7B forms dimeric configuration in the TGN, however, deleting the four MBDs from the N-terminus reshuffles the protein to the secretary vesicles, suggesting that the N-MBDs may also serve as a Golgi-retention signal [43]. These observations demonstrated the structural complexity involved in intramolecular transfer of metals by the mammalian ATPases.

Work on the bacterial system suggests that Cu chaperones can deliver Cu ions to TM-MBDs. Upon enzyme phosphorylation, Cu(I) is processed within the transmembrane region. Following the opening of the TM-MBD, Cu(I) is then released into the vesicles [51], which then traffic to the cellular membrane where Cu(I) is exported [52]. It was demonstrated that hydrolysis of one ATP molecule is sufficient to confer one molecule of Cu(I) translocation by the microbial ortholog CopA [51]. However, it is currently unclear whether this holds true for the mammalian Cu-ATPases.

Evidence has accumulated indicating that Pt(II) shares substantial similarity in coordination chemistry with Cu(I). The Cu-binding CXXC-motifs in Atox1 and ATP7A/ATP7B are also involved in cDDP binding [53]. Binding of cDDP to these sites may [54] or may not [55] displace Cu(I) binding. Mechanistic details of cDDP translocation in ATP7A/ATP7B is largely unknown because cellular levels of these ATPases are very low. Using microsomal fraction enriched in recombinant ATP7B (or ATP7A) absorbed onto a solid supported membrane, it was demonstrated that Pt drugs activate Cu-ATPases following a mechanism analogous to that of Cu(I) [54]. This in vitro study supports the roles of ATP7A and ATP7B in Pt drug transport. It has been reported that deletion of Atox1 resulted in an inability of cDDP delivery to ATP7A/ATP7B [37,38] and overexpression of ATP7A and ATP7B, resulting in impaired cDDP elimination, consequently resulting in cellular resistance to cDDP [56,57,58,59], Cbp [60], and Oxl [61].

## 3. Modulating cDDP Sensitivity through Redox Regulation of Cu Homeostasis

**Figure 2:**
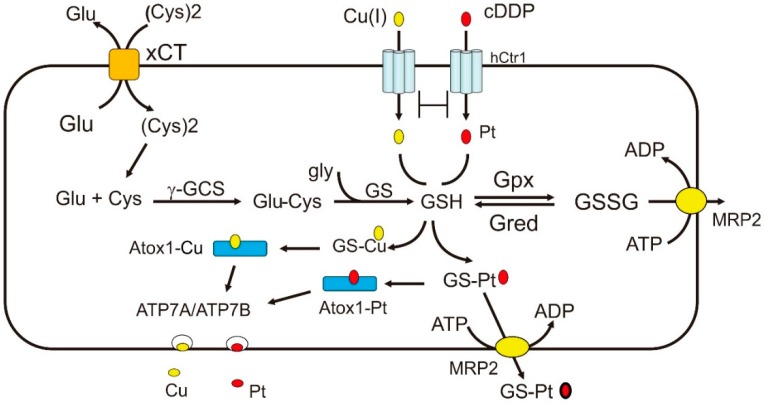
Regulation of Cu(I) and cDDP transports by the redox mechanism. The biosynthesis of GSH which plays important roles in redox regulation of Cu(I) and cDDP transport is shown here. The substrates of **γ**GCS are glutamine and cysteine which are transported by xCT. GSH is oxidized to GSSG by Gpx and GSSG is reduced back to GSH by Gred. GSH can facilitate Cu(I) and Pt(II) delivery to Atox1 and ATP7A/ATP7B. GSH can upregulate *hCtr1* expression because of its chelation with Cu(I). GSSG and Pt-GS conjugate can be transported by the MRP2 efflux pump. Abbreviations: xCT, cysteine-glutamine anti-polar transporter; Gpx, glutathione peroxidase; GS, glutathione synthetase; **γ**GCS, **γ**-glutamylcysteine synthetase; Gred, glutathione reductase; MRP2, multidrug resistance protein 2.

One of the important hallmarks in Pt-based chemotherapy is the induction of reactive oxygen species (ROS)-related oxidative stress that leads to cell killing [62]. It has been abundantly demonstrated that cDDP treatment induces expression of redox-regulating enzymes that are involved in the biosynthesis of glutathione (GSH), such as γ-glutamylcysteine synthetase (**γ**GCS) and glutathione synthetase (GS) (see review in [62] and references therein). GSH is an abundant cellular redox regulator (in mM quantity) (see reviewers in [63,64]) (Figure 2). cDDP-resistant cell lines established by long-term exposure to cDDP display elevated expression of these enzymes, and depleting GSH using buthionine sulfoximine (BSO) reverses cDDP resistance [62,63,65]. Glutamate (Glu) and cysteine (Cys) are the upstream substrates of GSH biosynthesis, and their cellular transports are carried out by the xCT cysteine-glutamate antiporter (Figure 2). Pharmacological inhibition of xCT increases cDDP sensitivity [66]. cDDP-resistant cells also show increased glutamine (Gln) uptake, which is the precursor of Glu biosynthesis [67]. Moreover, Gln-fed rats reduced cDDP-induced nephrotoxicity in the proximal tubules [68]. These results, collectively, support the important roles of the GSH biosynthetic system in cDDP resistance.

However, we demonstrated that increased GSH by transfection with recombinant DNA encoding **γ**GCS confers *sensitization*, but not resistance, to cDDP’s cell killing [64,65]. **γ**GCS is the rate-limiting enzyme for the biosynthesis of GSH. These findings demonstrate that elevated GSH does not per se confer cDDP resistance. We reason that the elevated expression of GSH and its biosynthetic enzymes observed in the cDDP-resistant cells under long-term cDDP exposure are likely due to CDDP-associated oxidative stress because these enzymes are redox-regulated. Instead, sensitization of the **γ**GCS-transfected cells to cDDP is due to alternation of *hCtr1* expression. In another study, Franzini et al. [69] reported that overexpression of γ-glutamyltransferase (GGT) exhibited reduced GSH levels in the transfected cells which displayed increased resistance to cDDP. No description of the expression of *hCtr1* was mentioned in this work, because *hCtr1* as a cDDP transporter was reported in the subsequent year [70]. We will discuss this further in light of GSH regulation of Cu(I)/cDDP transport below.

The redox system plays multiple roles in regulating Cu(I)/cDDP transport activities. First, extracellular Cu is normally present in an oxidized Cu(II) form. It is reduced to Cu(I) by cell membrane-associated reductases for the *hCtr1*, Atox1, and ATP7A/ATP7B transport cascade. Although Cu(I) can be converted into Cu(II) under oxidative stress conditions by the Fenton reaction, Cu(II) cannot be transported by these proteins. Second, Cu(I) and cDDP can bind to the major redox regulator, GSH and metallothionine (MT), resulting in reduction of bioavailability of Cu(I) that leads to increased *hCtr1* expression (see below). This can explain why GCS-transfection confers cDDP sensitivity [64,65]. Chelation of GSH by Cu(I) or Pt(II) induce GSH depletion, resulting in increased reactive oxygen species (ROS) production, which are generated from complex III in the mitochondria. The resultant ROS can modulate the expression of a whole spectrum of redox-regulating enzymes, including GSH peroxidase (Gpx) which converts GSH into GSSG, an oxidized form of GSH [63]. GSSG can be eliminated by the multidrug resistant protein transporter MRP2 (Figure 2). Third, the cytochrome c oxidase Cu chaperone (Cox17), which delivers Cu(I) to mitochondrial cytochrome c oxidase (CCO), can also deliver cDDP to mitochondria, the powerhouse of ROS production [71]. Platination of Cox17 affects the function of CCO, an important enzyme in the mitochondrial respiratory chain. Fourth, GSH can assist the conjugation of Cu(I) and Pt(II) with their chaperones and facilitate intracellular translocations of these metal ions. While apoAtox1 does not bind Cu-ATPases and is normally in monomeric configuration, Cu-Atox1 often exists as a dimer and its formation is strictly influenced by cellular redox conditions [72,73]. Under physiologic redox conditions, conjugation of Cu(I) and GSH exits as a polymer, which assists Cu(I) transferring to Atox1. GSH can also assist cDDP complex formation with Atox1. Moreover, Cu(I)-loaded Atox1 promotes the binding of cDDP and form the Atox1-Cu-Pt tertiary complex via sulfur-bridge linkages [74,75]. Likewise, GSH can facilitate the formation of Pt-Cox17 [71]. These findings demonstrate the important roles of GSH in Cu(I) and cDDP transport systems. Finally, Pt-(GSH)_2_ conjugate is also a known substrate of MRP2 efflux pump [76,77] (Figure 2), and elevated expression of MRP2 is associated with cDDP resistance [78].

## 4. Modulating cDDP Sensitivity through Transcriptional Regulation of *hCtr1* Expression

### 4.1. Regulation of Ctr1 Internalization by Cu Bioavailability

The findings that cDDP highjacks the Cu-transport system for its own transport underscore the importance of Cu transporters in regulating cDDP sensitivity in cancer chemotherapy. Homeostatic regulation of Ctr1 expression is an evolutionarily conserved mechanism from yeast [79] to humans [3,80,81]. Levels of Ctr1 are induced under Cu starvation conditions but are downregulated by Cu overload. A previous study in the yeast *Saccharomyces cerevisiae* demonstrated that Cu-induced yCtr1 internalization is one mechanism of Cu(I) acquisition, and that the internalized yCtr1 is rapidly degraded [82]. Cu-induced *hCtr1* internalization has also been reported in human cells [83,84]. However, detailed mechanisms on how mobilization of Ctr1 movements in response to Cu availability remain to be investigated.

### 4.2. Transcriptional Regulation of Ctr1 Expression by Cu Bioavailability

Transcriptional regulation of Ctr1 involves a variety of transcription factors. These transcription regulators generally contain Cu-sensing domains which bear DNA binding activities and transactivation domains for transcriptional activation. Budding yeast Mac1 is the transcriptional regulator controlling the expression of *yCtr1* and *yCtr3* and reductase *Fre1*, together encoding the Cu transport system. The N-terminal 40 amino acids of Mac1 contains zinc finger (ZF)-like motifs which have DNA-binding activities. Two cysteine (C)-rich transactivation domains are located at its C-terminus. Under low Cu conditions, Mac1 activates the expression of Cu transport genes by binding to the promoters of these genes. At high Cu concentrations, four Cu(I) ions each bind the C-rich transactivation domains, inducing conformational changes of Mac1, resulting in its fall-off from the target genes and shutting down of transcription [85]. In the meantime, another transcriptional factor, Ace1, is activated through the formation of a tetracopper-thiolate cluster within the Cu regulatory domain [86,87], and binds the promoter of *CUP1* and *CRS5* encoding Cu-chelating metallothionein (MT) [88]. Thus, yeasts use Mac1 and Ace1 in coordinating their activation and inactivation in response to Cu depletion and repletion conditions, respectively, to regulate Cu homeostasis.

**Figure 3:**
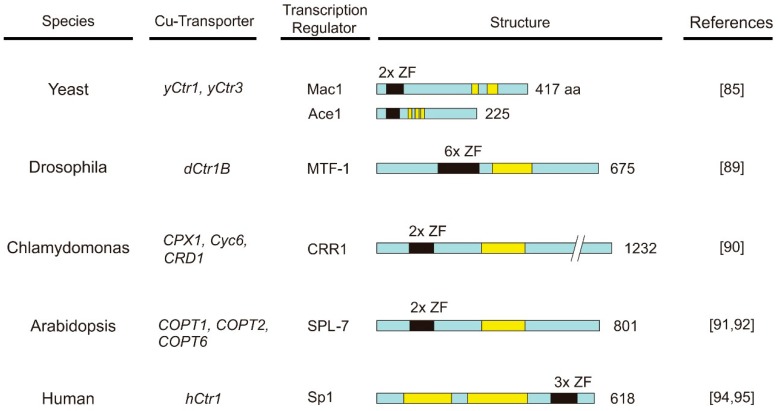
Schematic diagrams showing the structures of transcription factors for copper transporters from different species. Black boxes refer to ZF-like domains; yellow boxes, transactivation domains (see the text for details).

Zinc finger-like transcriptional factors are also involved in regulating *Ctr1* gene expression in other organisms. These include metal responsive transcription factor 1 (MTF-1) for the *dCtr1B* in *Drosophila* [89], CRR1 for the *Chlamydomonas* Cu transporters *Cyc6*, *CPX1*, and *CRD1* [90], and SQUAMOS promoter-binding protein like-7 (SPL-7) factor for three *Arabidopsis* Cu transporters, COPT1, COPT2 and COPT6 [91,92]. The schematic structure of transcription factors for *Ctr1* genes in different species is shown in Figure 3 [93].

**Figure 4:**
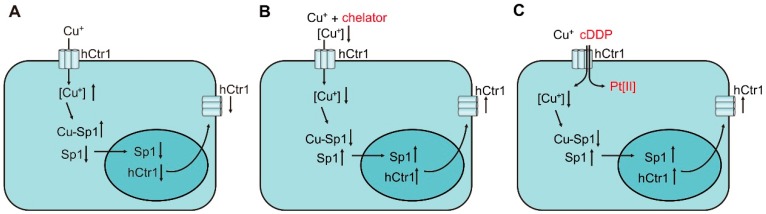
Mechanisms of transcriptional regulation of Sp1 and *hCtr1* in response to various challenges: (**A**) downregulation by Cu overload; (**B**) upregulation by Cu chelator; (**C**) upregulation by Cu(I) and cDDP combination.

In humans, we previously identified that specific protein (Sp1) is the transcription factor that regulates *hCtr1* expression [94,95]. Sp1 by itself is regulated by Cu homeostasis via transcriptional interactions with at least 10 Sp1-binding sites located at the Sp1 promoter. High Cu conditions downregulate Sp1 expression (Figure 4A), whereas reduced Cu conditions by Cu chelation upregulate Sp1 (Figure 4B). Sp1 in turn regulates the expression of *hCtr1* in response to Cu concentration variations accordingly (Figure 4A,B) by interacting with two Sp1-binding sites located 25 bp downstream from the *hCtr1* transcriptional start site. Systemic mutations of these Sp1-binding sites abolish the Cu responsiveness of Sp1 and *hCtr1* expression [95].

Another transcriptional unit with opposite direction, named *FKBP133* (also known *KIAA0674*) encodes an FK506-binding protein-like transcript, and is located −201 bp upstream of the *hCtr1* locus. FKBP133 is also regulated by Cu stressed conditions using the same Sp1 binding sites for the *hCtr1* regulation (our unpublished result). How this Sp1-mediated bidirectional transcription and the fine-turning of its targeted gene expression under Cu stressed conditions remains to be investigated.

### 4.3. The Sensing Mechanisms of Cu Bioavailability by Sp1

Sp1 is a ubiquitous transcription factor consisting of a DNA-binding domain at the C-terminus that contains three ZF and a transactivation domain that contains two serine/threonine-rich and two glutamine-rich (Q-rich 1 and Q-rich 2) subdomains (Figure 3). The ZF of Sp1 is constitutively bound by Zn(II) because apoSp1 is very unstable [96]. Each ZF consists of Cys2-His2 residues that is coordinated by one Zn(II) molecule. Elevated Cu ions displace Zn(II) binding of Sp1 [97]. Although this causes only minor structural alternations, the “Cu-finger” cannot interact with the *hCtr1* promoter [98]. Thus, Cu is a negative regulator of Sp1 by poisoning its ZF DNA binding domains. 

Sp1 is a member of the Sp/KLF (Krüppel-like factor) transcription factor family sharing the general three copies of C2H2-type ZFs [99]. However, ZF domains in this family have a variety of structures and at least eight different topologies have been categorized; many of these ZF-binding proteins do not respond to Cu challenges [100]. Even Sp3, which is the closest member of Sp1, does not regulate *hCtr1* expression [94]. 

Interaction between cDDP and Sp1 is very weak at best, although it can interact with other ZF-containing proteins, i.e., the retroviral protein NCp7 [101] and DNA polymerase 1 [102]. However, it has been reported that the reactivity of Pt(II) with ZFs can be modulated by reducing agents such as tris(2-carboxyethyl)phosphine) (TCEP) [103] which is commonly used to maintain cysteine residues at the reduced state [102]. In contrast, the *trans*-platinum thiazole Pt complex [PtCl2(NH3)(thiazole)] is highly reactive towards Sp1-ZF2, but the resulting Pt-thiazole complex prevents nuclear trafficking of Sp1 [103]. Moreover, the reactivity of Sp1-ZF3 with the cDDP complex is increased when the NH3 ligands of cDDP are replaced by the chelating ethylenediamine (en) in [PtCl_2_(en)] [104]. These reactions cause conformational distortion of ZFs, rendering it unable to bind DNA. These results illustrate the capacity for interaction of Pt compounds with ZF-proteins. However, the pharmacological relevance of the interaction of Pt compounds with ZF proteins remains to be further studied. 

We previously demonstrated that cDDP can transcriptionally induce Sp1 and *hCtr1* expression in time- and concentration-dependent manners [105]. Since cDDP does not directly act upon Sp1, these results can be interpreted by virtue that cDDP acts as a competitor for *hCtr1*-mediated Cu(I) transport, resulting in reduced cellular Cu levels that leads to upregulation of Sp1/*hCtr1* (Figure 4C). In this context, cDDP may be considered as a Cu-lowering agent, further supporting the integral role of cDDP in Cu homeostasis regulation.

### 4.4. The Capacity of hCtr1 Regulation and Cellular Cu Bioavailability

Copper is an essential micronutrient for growth but is toxic when in excess. This is evidenced by the findings that ablation of both murine *mCtr1* alleles results in embryonic lethality and the associated toxic effects in Menkes’ and Wilson’s diseases due to Cu anomalies in the intestine and liver, respectively, as mentioned above. It has been reported that almost all cellular Cu(I) are chelated by cellular constituents and only a small fraction of Cu(I) is bioavailable [106]. Thus, the bioavailable Cu pools have to be tightly regulated. While a yeast (*S. cerevisiae*) cell contains as high as 0.01–0.1 M of total Cu ions, but the bioavailable Cu(I) concentration is estimated to be only in the range of 8.9 × 10^−17^ to 5.1 × 10^−23^ M. This was estimated using the highly Cu(I)-selective and -sensitive transcription factors Mac1 and Ace1 for the lower and upper limits, respectively [107]. Estimation of bioavailable Cu(I) pools in human cells cannot be similarly carried out using Sp1 as a probe because of its abundant target genes. 

It is conceivable that bioavailable Cu(I) pools are different from cell type to cell type and play a critical role in determining the capacity of Cu homeostasis regulation under Cu stressed conditions. Intestinal epithelium is the primary cell type acquiring Cu from food. Intestinal epithelial cell-specific knockout mice show drastic reduction of Cu in different organs, i.e., about 95% in the liver but only about 30% in the kidneys as compared respectively with those in normal mice [108], refracting differential capacities of Cu homeostatic regulation in different tissues.

A great variety of human malignancies have shown elevated Cu levels in the serum and tumors, and elevated Cu levels are often positively correlated with cancer progression (see review in [109] and references therein). Elevated Cu levels in tumors are correlated with high levels of *hCtr1* in lung cancers [110]. Likewise, Sp1 expression levels are elevated in many human tumors, including tumors of stomach, pancreas, breast, brain, and thyroid (see [111] and references therein). These findings suggest that capacity of Cu bioavailability regulation may differ between human cancers and their normal counterparts.

## 5. Modulation of *hCtr1* Transcriptional Regulation for Overcoming cDDP Resistance in Cancer Chemotherapy

By analyzing a publicly available database containing 91 ovarian cancers, Ishida et al. [70] reported that patients with high *hCtr1* levels were associated with longer disease-free survival time after adjuvant chemotherapy with a Pt drug and taxane. We analyzed a database of 243 patients with endometrioid tumors of the ovary treated with first line Pt/taxane chemotherapy and found that patients with high *hCtr1* levels, but not ATP7A and ATP7B, have significantly longer progression-free survival (PFS) and OS than those with low *hCtr1* [20]. Recently, a more comprehensive study based on eight datasets containing 2149 patients from nine countries, Sun et al. [8] reported that high *hCtr1* expression is associated with favorable treatment outcomes in ovarian and lung cancer patients who underwent Pt-based chemotherapy. These results suggest that cancers with high *hCtr1* expression have a better response to Pt-based drugs, suggesting that transcriptional upregulation of *hCtr1* expression by Cu-lowering agents can be an attractive strategy for improving chemosensitivity to Pt drugs with the following considerations. Firstly, we demonstrated in a cultured cell study that *hCtr1* mRNA and protein levels can be induced by Cu chelators within one hour, but it takes several days for the upregulated *hCtr1* mRNA and protein to return to basal levels upon removal of the chelators. These results suggest that induced *hCtr1* by Cu chelation is considerably stable. Neither ATP7A nor ATP7B mRNA is altered under Cu chelation [112]. Secondly, as stated, while multiple mechanisms are involved cDDP resistance, reduced *hCtr1* expression is a common mechanism. Moreover, we found that cDDP-resistant cancer cells exhibit a greater magnitude of *hCtr1* upregulation by Cu-lowering agents as compared with their drug-sensitive counterparts. These results were demonstrated in three independent cDDP-resistant cell lines treated with three different Cu-lowering agents, i.e., trientine, D-pencillamide (D-pen), and tetrathiomolybdate (TM). The reduced levels of *hCtr1* in these cDDP-resistant cells were recovered to those comparable with the corresponding drug-sensitive counterparts. The effectiveness of these Cu-lowering agents have little cell line- and agent-specificities [112]. These findings demonstrate that Cu-lowering agents can overcome cDDP resistance [112]. Third, Cu-lowering agents by themselves have been in many clinical trials for treating various human malignancies because Cu is involved in tumor growth pathways, such as metastatic tumor angiogenesis [113] and the oncogenic transformation pathway [114,115]. 

Based on these pre-clinical observations, a phase I clinical trial using Cbp plus trientine in 55 patients with advanced malignancies, 45 of which had prior failure in Pt drug treatment, was conducted at MD Anderson Cancer. About 19% of patients (*n* = 9) who maintained low serum Cu levels after the treatments had significantly longer median PFS (*p* = 0.001) and OS (*p* = 0.03) as compared with those patients (*n* = 38) who did not [116,117]. However, while the response rate remains low—given the heterogeneity of patient population and multiple mechanisms of drug resistance that may be involved, and the intrinsic variation in the capacity of *hCtr1* induction by Cu-lowering agent as mentioned above—there are options for improvement using this strategy (see below).

## 6. Conclusions and Perspectives

Pt drugs have been effective in treating many human malignancies, however, treatment efficacy has been hampered by drug resistance. Mechanisms of cDDP resistance are complex, and defective drug transport is commonly associated with cDDP resistance [63,118,119]. Recent studies have established that the Pt drug transport system is an integral mechanism of Cu homeostasis regulation. Preclinical studies have established that interventions of expression of *hCtr1* transporter, Atox1 chaperone, and ATP7A/ATP7B efflux pumps can affect chemosensitivity of Pt drugs. However, targeting this transport system for overcoming cDDP resistance has not been translated into a therapeutic benefit. Our laboratories have discovered that *hCtr1*, but not ATP7A/ATP7B, expression can be transcriptionally modulated by Cu concentration variations via the transcriptional factor Sp1. The ZF domains of Sp1 are sensors of both Cu repletion and depletion conditions and transcriptionally down- and up-regulates Sp1 accordingly. Sp1 in turn regulates *hCtr1* expression, which is the major regulator of cellular Cu content. This constitutes the Cu-Sp1-*hCtr1* homeostatic self-regulatory loop [3,120]. 

Not only does *hCtr1* function as an important importer for cellular cDDP accumulation, its expression is also upregulated by cDDP, because cDDP functions as a potent competitor for *hCtr1*-mediated Cu transport, resulting in reduced intracellular Cu content. These observations have led to an exploratory clinical investigation using the Cu-lowering agent trientine to enhance *hCtr1* expression for overcoming Pt resistance in multiple cancer types [116]. Although the outcome of this first-in-human trial remains low, the established mechanistic basis has important potentials for further clinical studies in improving the treatment efficacy of Pt-based chemotherapy, with the following considerations. 

Firstly, the ZF transcription regulators have been firmly assessed in Cu sensing to transcriptionally regulate Pt drug transporters in eukaryotic cells. There are 23,299 genes encoding ZF-containing proteins in the human genome [121]. This provides a vast wealth of opportunities for exploring other ZF regulators that may control the cellular concentration of Pt drugs. These ZF proteins may directly regulate the expression of Pt transporters, or indirectly function as intracellular metal chelators that regulate global Cu ion bioavailability which regulates the expression of the Pt transport system. Secondly, given the observations that Ctr1 expression is tissue-specific, and its responses to Cu depletion differs in different organs [108], it is important to elucidate tissue-specific mechanisms of *hCtr1* expression using the Cu-chelation strategy. This research may identify specific tumor types that are favorable for Cu chelation therapy. As an example, we found that expression of *hCtr1* mRNA and proteins are higher in 20 out of 20 ovarian tumor biopsies as compared with their adjacent naïve tissues in patients prior to chemotherapy (HHWC, unpublished data). These results, together with those published previously [8,70,112], may explain why ovarian cancers are preferentially sensitive to Pt-based chemotherapy [122,123], and why cDDP-refractory ovarian cancers may seem to have better response rates to the trientine plus Cbp combination therapy than other tumors in our exploratory clinical investigation [117]. Thirdly, previous clinical studies demonstrated that better treatment outcomes are associated in patients with reduced serum Cu levels after the trientine/Cbp combination therapy [116,117]. These results suggest that effective therapy may lie in the Cu-lowering ability of the treatment. Thus, identification of predictive biomarkers for Cu chelation in patients may be critical. Fourthly, the current clinical study used trientine; the effectiveness of other potent Cu-lowering agents such as desferal [124] and TM [125] still need to be explored. Fifthly, cytotoxic effects associated with combination therapy using a Pt drug and copper-lowering agent need to be critically evaluated. Finally, while we have learned much about *hCtr1* regulation, other transport components such as Atox1 and ATP7B/ATP7B remains a largely uncharted area of research. Exploitation of drug retention by targeting these transporters may be fruitful. 

In summary, we have highlighted the importance of targeting drug transporters in this review. The described transcriptional regulation of *hCtr1* presented here may serve as the translational basis for future investigations for improving the treatment efficacy of cancer chemotherapy using Pt-based drugs. 

## Abbreviations

Atox1	antioxidant 1
BSO	buthionine sulfoximine
Cbp	carboplatin
Ctr1	the high-affinity copper transporter 1 (SLC31A1)
GCS	γ-glutamylcysteine synthetase
GGT	γ-glutamyltransferase
GS	glutathione synthetase
GSH	Glutathione
MTF-1	metal responsive transcription factor 1
D-pen	D-pencillamide
Oxl	oxaliplatin
ROS	reactive oxygen species
Sp1	specific protein
TM	tetrathiomolybdate
TGN	trans-Golgi network
xCT	cystine glutamate antipolar transporter

## References

[1] Muggia F.M., Bonetti A., Hoeschele J.D., Rozencweig M., Howell S.B. (2015). Platinum Antitumor Complexes: 50 Years Since Barnett Rosenberg’s Discovery. J. Clin. Oncol..

[2] Johnstone T.C., Suntharalingam K., Lippard S.J. (2016). The Next Generation of Platinum Drugs: Targeted Pt(II) Agents, Nanoparticle Delivery, and Pt(IV) Prodrugs. Chem. Rev..

[3] Chen H.H., Chen W.C., Liang Z.D., Tsai W.B., Long Y., Aiba I., Fu S., Broaddus R., Liu J., Feun L.G. (2015). Targeting drug transport mechanisms for improving platinum-based cancer chemotherapy. Expert Opin. Ther. Targets.

[4] Gatti L., Cassinelli G., Zaffaroni N., Lanzi C., Perego P. (2015). New mechanisms for old drugs: Insights into DNA-unrelated effects of platinum compounds and drug resistance determinants. Drug Resist. Updates.

[5] Kim E.S., Lee J.J., He G., Chow C.W., Fujimoto J., Kalhor N., Swisher S.G., Wistuba I.I., Stewart D.J., Siddik Z.H. (2012). Tissue platinum concentration and tumor response in non-small-cell lung cancer. J. Clin. Oncol..

[6] Hall M.D., Okabe M., Shen D.W., Liang X.J., Gottesman M.M. (2008). The role of cellular accumulation in determining sensitivity to platinum-based chemotherapy. Annu. Rev. Pharmacol. Toxicol..

[7] Andrews P.A., Howell S.B. (1990). Cellular pharmacology of cisplatin: Perspectives on mechanisms of acquired resistance. Cancer Cells.

[8] Sun S., Cai J., Yang Q., Zhao S., Wang Z. (2017). The association between copper transporters and the prognosis of cancer patients undergoing chemotherapy: A meta-analysis of literatures and datasets. Oncotarget.

[9] Long Y., Tsai W.B., Chang J.T., Estecio M., Wangpaichitr M., Savaraj N., Feun L.G., Chen H.H., Kuo M.T. (2016). Cisplatin-induced synthetic lethality to arginine-starvation therapy by transcriptional suppression of ASS1 is regulated by DEC1, HIF-1alpha, and c-Myc transcription network and is independent of ASS1 promoter DNA methylation. Oncotarget.

[10] Gately D.P., Howell S.B. (1993). Cellular accumulation of the anticancer agent cisplatin: A review. Br. J. Cancer.

[11] Ivy K.D., Kaplan J.H. (2013). A re-evaluation of the role of *hCtr1*, the human high-affinity copper transporter, in platinum-drug entry into human cells. Mol. Pharmacol..

[12] Ohrvik H., Thiele D.J. (2015). The role of Ctr1 and Ctr2 in mammalian copper homeostasis and platinum-based chemotherapy. J. Trace Elem. Med. Biol..

[13] Martelli L., Di Mario F., Ragazzi E., Apostoli P., Leone R., Perego P., Fumagalli G. (2006). Different accumulation of cisplatin, oxaliplatin and JM216 in sensitive and cisplatin-resistant human cervical tumour cells. Biochem. Pharmacol..

[14] Buss I., Hamacher A., Sarin N., Kassack M.U., Kalayda G.V. (2018). Relevance of copper transporter 1 and organic cation transporters 1–3 for oxaliplatin uptake and drug resistance in colorectal cancer cells. Metall. Integr. Biomet. Sci..

[15] Sprowl J.A., Ciarimboli G., Lancaster C.S., Giovinazzo H., Gibson A.A., Du G., Janke L.J., Cavaletti G., Shields A.F., Sparreboom A. (2013). Oxaliplatin-induced neurotoxicity is dependent on the organic cation transporter OCT2. Proc. Natl. Acad. Sci. USA.

[16] Ishida S., Lee J., Thiele D.J., Herskowitz I. (2002). Uptake of the anticancer drug cisplatin mediated by the copper transporter Ctr1 in yeast and mammals. Proc. Natl. Acad. Sci. USA.

[17] Song I.S., Savaraj N., Siddik Z.H., Liu P., Wei Y., Wu C.J., Kuo M.T. (2004). Role of human copper transporter Ctr1 in the transport of platinum-based antitumor agents in cisplatin-sensitive and cisplatin-resistant cells. Mol. Cancer Ther..

[18] Beretta G.L., Gatti L., Tinelli S., Corna E., Colangelo D., Zunino F., Perego P. (2004). Cellular pharmacology of cisplatin in relation to the expression of human copper transporter CTR1 in different pairs of cisplatin-sensitive and -resistant cells. Biochem. Pharmacol..

[19] Rabik C.A., Maryon E.B., Kasza K., Shafer J.T., Bartnik C.M., Dolan M.E. (2009). Role of copper transporters in resistance to platinating agents. Cancer Chemother. Pharmacol..

[20] Liang Z.D., Stockton D., Savaraj N., Tien Kuo M. (2009). Mechanistic comparison of human high-affinity copper transporter 1-mediated transport between copper ion and cisplatin. Mol. Pharmacol..

[21] Du X., Wang X., Li H., Sun H. (2012). Comparison between copper and cisplatin transport mediated by human copper transporter 1 (*hCtr1*). Metall. Integr. Biomet. Sci..

[22] Aller S.G., Unger V.M. (2006). Projection structure of the human copper transporter CTR1 at 6-A resolution reveals a compact trimer with a novel channel-like architecture. Proc. Natl. Acad. Sci. USA.

[23] Pottier A., Borghi E., Levy L. (2014). New use of metals as nanosized radioenhancers. Anticancer Res..

[24] Logeman B.L., Wood L.K., Lee J., Thiele D.J. (2017). Gene duplication and neo-functionalization in the evolutionary and functional divergence of the metazoan copper transporters Ctr1 and Ctr2. J. Biol. Chem..

[25] Ohrvik H., Nose Y., Wood L.K., Kim B.E., Gleber S.C., Ralle M., Thiele D.J. (2013). Ctr2 regulates biogenesis of a cleaved form of mammalian Ctr1 metal transporter lacking the copper- and cisplatin-binding ecto-domain. Proc. Natl. Acad. Sci. USA.

[26] Ohrvik H., Logeman B., Turk B., Reinheckel T., Thiele D.J. (2016). Cathepsin Protease Controls Copper and Cisplatin Accumulation via Cleavage of the Ctr1 Metal-binding Ectodomain. J. Biol. Chem..

[27] Bompiani K.M., Tsai C.Y., Achatz F.P., Liebig J.K., Howell S.B. (2016). Copper transporters and chaperones CTR1, CTR2, ATOX1, and CCS as determinants of cisplatin sensitivity. Metall. Integr. Biomet. Sci..

[28] Lee Y.Y., Choi C.H., Do I.G., Song S.Y., Lee W., Park H.S., Song T.J., Kim M.K., Kim T.J., Lee J.W. (2011). Prognostic value of the copper transporters, CTR1 and CTR2, in patients with ovarian carcinoma receiving platinum-based chemotherapy. Gynecol. Oncol..

[29] Yoshida H., Teramae M., Yamauchi M., Fukuda T., Yasui T., Sumi T., Honda K., Ishiko O. (2013). Association of copper transporter expression with platinum resistance in epithelial ovarian cancer. Anticancer Res..

[30] Robinson N.J., Winge D.R. (2010). Copper metallochaperones. Annu. Rev. Biochem..

[31] Flores A.G., Unger V.M. (2013). Atox1 contains positive residues that mediate membrane association and aid subsequent copper loading. J. Membr. Biol..

[32] Kahra D., Kovermann M., Wittung-Stafshede P. (2016). The C-Terminus of Human Copper Importer Ctr1 Acts as a Binding Site and Transfers Copper to Atox1. Biophys. J..

[33] Wu X., Yuan S., Wang E., Tong Y., Ma G., Wei K., Liu Y. (2017). Platinum transfer from *hCtr1* to Atox1 is dependent on the type of platinum complex. Metall. Integr. Biomet. Sci..

[34] Banci L., Bertini I., Ciofi-Baffoni S., Kozyreva T., Zovo K., Palumaa P. (2010). Affinity gradients drive copper to cellular destinations. Nature.

[35] Wernimont A.K., Huffman D.L., Lamb A.L., O'Halloran T.V., Rosenzweig A.C. (2000). Structural basis for copper transfer by the metallochaperone for the Menkes/Wilson disease proteins. Nat. Struct. Biol..

[36] Boal A.K., Rosenzweig A.C. (2009). Crystal structures of cisplatin bound to a human copper chaperone. J. Am. Chem. Soc..

[37] Hua H., Gunther V., Georgiev O., Schaffner W. (2011). Distorted copper homeostasis with decreased sensitivity to cisplatin upon chaperone Atox1 deletion in Drosophila. Biometals.

[38] Safaei R., Maktabi M.H., Blair B.G., Larson C.A., Howell S.B. (2009). Effects of the loss of Atox1 on the cellular pharmacology of cisplatin. J. Inorg. Biochem..

[39] Itoh S., Kim H.W., Nakagawa O., Ozumi K., Lessner S.M., Aoki H., Akram K., McKinney R.D., Ushio-Fukai M., Fukai T. (2008). Novel role of antioxidant-1 (Atox1) as a copper-dependent transcription factor involved in cell proliferation. J. Biol. Chem..

[40] Itoh S., Ozumi K., Kim H.W., Nakagawa O., McKinney R.D., Folz R.J., Zelko I.N., Ushio-Fukai M., Fukai T. (2009). Novel mechanism for regulation of extracellular SOD transcription and activity by copper: Role of antioxidant-1. Free Radic. Biol. Med..

[41] Celauro E., Mukaj A., Fierro-Gonzalez J.C., Wittung-Stafshede P. (2017). Copper chaperone ATOX1 regulates pluripotency factor OCT4 in preimplantation mouse embryos. Biochem. Biophys. Res. Commun..

[42] Inesi G., Pilankatta R., Tadini-Buoninsegni F. (2014). Biochemical characterization of P-type copper ATPases. Biochem. J..

[43] Jayakanthan S., Braiterman L.T., Hasan N.M., Unger V.M., Lutsenko S. (2017). Human Copper Transporter Atp7b (Wilson Disease Protein) Forms Stable Dimers in Vitro and in Cells. J. Biol. Chem..

[44] Cox D.W., Moore S.D. (2002). Copper transporting P-type ATPases and human disease. J. Bioenergy Biomembr..

[45] Lutsenko S., Barnes N.L., Bartee M.Y., Dmitriev O.Y. (2007). Function and regulation of human copper-transporting ATPases. Physiol. Rev..

[46] Arnesano F., Banci L., Bertini I., Thompsett A.R. (2002). Solution structure of CopC: A cupredoxin-like protein involved in copper homeostasis. Structure.

[47] Hussain F., Olson J.S., Wittung-Stafshede P. (2008). Conserved residues modulate copper release in human copper chaperone Atox1. Proc. Natl. Acad. Sci. USA.

[48] Xi Z., Shi C., Tian C., Liu Y. (2013). Conserved residue modulates copper-binding properties through structural dynamics in human copper chaperone Atox1. Metall. Integr. Biomet. Sci..

[49] Yu C.H., Yang N., Bothe J., Tonelli M., Nokhrin S., Dolgova N.V., Braiterman L., Lutsenko S., Dmitriev O.Y. (2017). The metal chaperone Atox1 regulates the activity of the human copper transporter ATP7B by modulating domain dynamics. J. Biol. Chem..

[50] Lewis D., Pilankatta R., Inesi G., Bartolommei G., Moncelli M.R., Tadini-Buoninsegni F. (2012). Distinctive features of catalytic and transport mechanisms in mammalian sarco-endoplasmic reticulum Ca^2+^ ATPase (SERCA) and Cu^+^ (ATP7A/B) ATPases. J. Biol. Chem..

[51] Gonzalez-Guerrero M., Arguello J.M. (2008). Mechanism of Cu+-transporting ATPases: Soluble Cu^+^ chaperones directly transfer Cu^+^ to transmembrane transport sites. Proc. Natl. Acad. Sci. USA.

[52] Pilankatta R., Lewis D., Inesi G. (2011). Involvement of protein kinase D in expression and trafficking of ATP7B (copper ATPase). J. Biol. Chem..

[53] Safaei R., Adams P.L., Maktabi M.H., Mathews R.A., Howell S.B. (2012). The CXXC motifs in the metal binding domains are required for ATP7B to mediate resistance to cisplatin. J. Inorg. Biochem..

[54] Tadini-Buoninsegni F., Bartolommei G., Moncelli M.R., Inesi G., Galliani A., Sinisi M., Losacco M., Natile G., Arnesano F. (2014). Translocation of platinum anticancer drugs by human copper ATPases ATP7A and ATP7B. Angew. Chem..

[55] Palm M.E., Weise C.F., Lundin C., Wingsle G., Nygren Y., Bjorn E., Naredi P., Wolf-Watz M., Wittung-Stafshede P. (2011). Cisplatin binds human copper chaperone Atox1 and promotes unfolding in vitro. Proc. Natl. Acad. Sci. USA.

[56] Nakagawa T., Inoue Y., Kodama H., Yamazaki H., Kawai K., Suemizu H., Masuda R., Iwazaki M., Yamada S., Ueyama Y. (2008). Expression of copper-transporting P-type adenosine triphosphatase (ATP7B) correlates with cisplatin resistance in human non-small cell lung cancer xenografts. Oncol. Rep..

[57] Komatsu M., Sumizawa T., Mutoh M., Chen Z.S., Terada K., Furukawa T., Yang X.L., Gao H., Miura N., Sugiyama T. (2000). Copper-transporting P-type adenosine triphosphatase (ATP7B) is associated with cisplatin resistance. Cancer Res..

[58] Safaei R., Howell S.B. (2005). Copper transporters regulate the cellular pharmacology and sensitivity to Pt drugs. Crit. Rev. Oncol. Hematol..

[59] Leonhardt K., Gebhardt R., Mossner J., Lutsenko S., Huster D. (2009). Functional interactions of Cu-ATPase ATP7B with cisplatin and the role of ATP7B in the resistance of cells to the drug. J. Biol. Chem..

[60] Samimi G., Safaei R., Katano K., Holzer A.K., Rochdi M., Tomioka M., Goodman M., Howell S.B. (2004). Increased expression of the copper efflux transporter ATP7A mediates resistance to cisplatin, carboplatin, and oxaliplatin in ovarian cancer cells. Clin. Cancer Res..

[61] Martinez-Balibrea E., Martinez-Cardus A., Musulen E., Gines A., Manzano J.L., Aranda E., Plasencia C., Neamati N., Abad A. (2009). Increased levels of copper efflux transporter ATP7B are associated with poor outcome in colorectal cancer patients receiving oxaliplatin-based chemotherapy. Int. J. Cancer.

[62] Brozovic A., Ambriovic-Ristov A., Osmak M. (2010). The relationship between cisplatin-induced reactive oxygen species, glutathione, and BCL-2 and resistance to cisplatin. Crit. Rev. Toxicol..

[63] Stewart D.J. (2007). Mechanisms of resistance to cisplatin and carboplatin. Crit. Rev. Oncol. Hematol..

[64] Chen H.H., Kuo M.T. (2010). Role of glutathione in the regulation of Cisplatin resistance in cancer chemotherapy. Met.-Based Drugs.

[65] Chen H.H., Song I.S., Hossain A., Choi M.K., Yamane Y., Liang Z.D., Lu J., Wu L.Y., Siddik Z.H., Klomp L.W. (2008). Elevated glutathione levels confer cellular sensitization to cisplatin toxicity by up-regulation of copper transporter *hCtr1*. Mol. Pharmacol..

[66] Roh J.L., Kim E.H., Jang H., Shin D. (2017). Aspirin plus sorafenib potentiates cisplatin cytotoxicity in resistant head and neck cancer cells through xCT inhibition. Free Radic. Biol. Med..

[67] Wangpaichitr M., Wu C., Li Y.Y., Nguyen D.J.M., Kandemir H., Shah S., Chen S., Feun L.G., Prince J.S., Kuo M.T. (2017). Exploiting ROS and metabolic differences to kill cisplatin resistant lung cancer. Oncotarget.

[68] Kim H.J., Park D.J., Kim J.H., Jeong E.Y., Jung M.H., Kim T.H., Yang J.I., Lee G.W., Chung H.J., Chang S.H. (2015). Glutamine protects against cisplatin-induced nephrotoxicity by decreasing cisplatin accumulation. J. Pharmacol. Sci..

[69] Franzini M., Corti A., Lorenzini E., Paolicchi A., Pompella A., De Cesare M., Perego P., Gatti L., Leone R., Apostoli P. (2006). Modulation of cell growth and cisplatin sensitivity by membrane gamma-glutamyltransferase in melanoma cells. Eur. J. Cancer.

[70] Ishida S., McCormick F., Smith-McCune K., Hanahan D. (2010). Enhancing tumor-specific uptake of the anticancer drug cisplatin with a copper chelator. Cancer Cell.

[71] Zhao L., Cheng Q., Wang Z., Xi Z., Xu D., Liu Y. (2014). Cisplatin binds to human copper chaperone Cox17: The mechanistic implication of drug delivery to mitochondria. Chem. Commun..

[72] Narindrasorasak S., Zhang X., Roberts E.A., Sarkar B. (2004). Comparative analysis of metal binding characteristics of copper chaperone proteins, Atx1 and ATOX1. Bioinorg. Chem. Appl..

[73] Tanchou V., Gas F., Urvoas A., Cougouluegne F., Ruat S., Averseng O., Quemeneur E. (2004). Copper-mediated homo-dimerisation for the HAH1 metallochaperone. Biochem. Biophys. Res. Commun..

[74] Dolgova N.V., Yu C., Cvitkovic J.P., Hodak M., Nienaber K.H., Summers K.L., Cotelesage J.J.H., Bernholc J., Kaminski G.A., Pickering I.J. (2017). Binding of Copper and Cisplatin to Atox1 Is Mediated by Glutathione through the Formation of Metal-Sulfur Clusters. Biochemistry.

[75] Xi Z., Guo W., Tian C., Wang F., Liu Y. (2013). Copper binding promotes the interaction of cisplatin with human copper chaperone Atox1. Chem. Commun..

[76] Ishikawa T. (1992). The ATP-dependent glutathione S-conjugate export pump. Trends Biochem. Sci..

[77] Ishikawa T., Ali-Osman F. (1993). Glutathione-associated cis-diamminedichloroplatinum(II) metabolism and ATP-dependent efflux from leukemia cells. Molecular characterization of glutathione-platinum complex and its biological significance. J. Biol. Chem..

[78] Yamasaki M., Makino T., Masuzawa T., Kurokawa Y., Miyata H., Takiguchi S., Nakajima K., Fujiwara Y., Matsuura N., Mori M. (2011). Role of multidrug resistance protein 2 (MRP2) in chemoresistance and clinical outcome in oesophageal squamous cell carcinoma. Br. J. Cancer.

[79] Dancis A., Yuan D.S., Haile D., Askwith C., Eide D., Moehle C., Kaplan J., Klausner R.D. (1994). Molecular characterization of a copper transport protein in *S. cerevisiae*: An unexpected role for copper in iron transport. Cell.

[80] Kuo M.T., Fu S., Savaraj N., Chen H.H. (2012). Role of the human high-affinity copper transporter in copper homeostasis regulation and cisplatin sensitivity in cancer chemotherapy. Cancer Res..

[81] Howell S.B., Safaei R., Larson C.A., Sailor M.J. (2010). Copper transporters and the cellular pharmacology of the platinum-containing cancer drugs. Mol. Pharmacol..

[82] Ooi C.E., Rabinovich E., Dancis A., Bonifacino J.S., Klausner R.D. (1996). Copper-dependent degradation of the Saccharomyces cerevisiae plasma membrane copper transporter Ctr1p in the apparent absence of endocytosis. EMBO J..

[83] Guo Y., Smith K., Lee J., Thiele D.J., Petris M.J. (2004). Identification of methionine-rich clusters that regulate copper-stimulated endocytosis of the human Ctr1 copper transporter. J. Biol. Chem..

[84] Molloy S.A., Kaplan J.H. (2009). Copper-dependent recycling of *hCtr1*, the human high affinity copper transporter. J. Biol. Chem..

[85] Jensen L.T., Posewitz M.C., Srinivasan C., Winge D.R. (1998). Mapping of the DNA binding domain of the copper-responsive transcription factor Mac1 from Saccharomyces cerevisiae. J. Biol. Chem..

[86] Furst P., Hu S., Hackett R., Hamer D. (1988). Copper activates metallothionein gene transcription by altering the conformation of a specific DNA binding protein. Cell.

[87] Keller G., Bird A., Winge D.R. (2005). Independent metalloregulation of Ace1 and Mac1 in Saccharomyces cerevisiae. Eukaryot Cell.

[88] Thiele D.J. (1988). ACE1 regulates expression of the Saccharomyces cerevisiae metallothionein gene. Mol. Cell. Biol..

[89] Selvaraj A., Balamurugan K., Yepiskoposyan H., Zhou H., Egli D., Georgiev O., Thiele D.J., Schaffner W. (2005). Metal-responsive transcription factor (MTF-1) handles both extremes, copper load and copper starvation, by activating different genes. Genes Dev..

[90] Strenkert D., Schmollinger S., Sommer F., Schulz-Raffelt M., Schroda M. (2011). Transcription factor-dependent chromatin remodeling at heat shock and copper-responsive promoters in Chlamydomonas reinhardtii. Plant Cell.

[91] Garcia-Molina A., Xing S., Huijser P. (2014). Functional characterisation of Arabidopsis SPL7 conserved protein domains suggests novel regulatory mechanisms in the Cu deficiency response. BMC Plant Biol..

[92] Yamasaki H., Hayashi M., Fukazawa M., Kobayashi Y., Shikanai T. (2009). SQUAMOSA Promoter Binding Protein-Like7 Is a Central Regulator for Copper Homeostasis in Arabidopsis. Plant Cell.

[93] Kuo M.T., Chen H.H. (2013). Overcoming platinum drug resistance with copper-lowering agents. Anticancer Res..

[94] Liang Z.D., Tsai W.B., Lee M.Y., Savaraj N., Kuo M.T. (2012). Specificity protein 1 (sp1) oscillation is involved in copper homeostasis maintenance by regulating human high-affinity copper transporter 1 expression. Mol. Pharmacol..

[95] Song I.S., Chen H.H., Aiba I., Hossain A., Liang Z.D., Klomp L.W., Kuo M.T. (2008). Transcription factor Sp1 plays an important role in the regulation of copper homeostasis in mammalian cells. Mol. Pharmacol..

[96] Bittel D.C., Smirnova I.V., Andrews G.K. (2000). Functional heterogeneity in the zinc fingers of metalloregulatory protein metal response element-binding transcription factor-1. J. Biol. Chem..

[97] Yan D., Aiba I., Chen H.H., Kuo M.T. (2016). Effects of Cu(II) and cisplatin on the stability of Specific protein 1 (Sp1)-DNA binding: Insights into the regulation of copper homeostasis and platinum drug transport. J. Inorg. Biochem..

[98] Yuan S., Chen S., Xi Z., Liu Y. (2017). Copper-finger protein of Sp1: The molecular basis of copper sensing. Metall. Integr. Biomet. Sci..

[99] Wierstra I. (2008). Sp1: Emerging roles—Beyond constitutive activation of TATA-less housekeeping genes. Biochem. Biophys. Res. Commun..

[100] Krishna S.S., Majumdar I., Grishin N.V. (2003). Structural classification of zinc fingers: Survey and summary. Nucleic Acids Res..

[101] Anzellotti A.I., Liu Q., Bloemink M.J., Scarsdale J.N., Farrell N. (2006). Targeting retroviral Zn finger-DNA interactions: A small-molecule approach using the electrophilic nature of trans-platinum-nucleobase compounds. Chem. Biol..

[102] Maurmann L., Bose R.N. (2010). Unwinding of zinc finger domain of DNA polymerase I by cis-diamminedichloroplatinum(II). Dalton Trans..

[103] Chen S., Xu D., Jiang H., Xi Z., Zhu P., Liu Y. (2012). Trans-platinum/thiazole complex interferes with Sp1 zinc-finger protein. Angew. Chem..

[104] Du Z., de Paiva R.E., Qu Y., Farrell N. (2016). Tuning the reactivity of Sp1 zinc fingers with platinum complexes. Dalton Trans..

[105] Liang Z.D., Long Y., Chen H.H., Savaraj N., Kuo M.T. (2014). Regulation of the high-affinity copper transporter (*hCtr1*) expression by cisplatin and heavy metals. J. Biol. Inorg. Chem..

[106] Rae T.D., Schmidt P.J., Pufahl R.A., Culotta V.C., O’Halloran T.V. (1999). Undetectable intracellular free copper: The requirement of a copper chaperone for superoxide dismutase. Science.

[107] Wegner S.V., Sun F., Hernandez N., He C. (2011). The tightly regulated copper window in yeast. Chem. Commun..

[108] Nose Y., Kim B.E., Thiele D.J. (2006). Ctr1 drives intestinal copper absorption and is essential for growth, iron metabolism, and neonatal cardiac function. Cell Metab..

[109] Gupte A., Mumper R.J. (2009). Elevated copper and oxidative stress in cancer cells as a target for cancer treatment. Cancer Treat. Rev..

[110] Kim E.S., Tang X., Peterson D.R., Kilari D., Chow C.W., Fujimoto J., Kalhor N., Swisher S.G., Stewart D.J., Wistuba I.I. (2014). Copper transporter CTR1 expression and tissue platinum concentration in non-small cell lung cancer. Lung Cancer.

[111] Beishline K., Azizkhan-Clifford J. (2015). Sp1 and the ‘hallmarks of cancer’. FEBS J..

[112] Liang Z.D., Long Y., Tsai W.B., Fu S., Kurzrock R., Gagea-Iurascu M., Zhang F., Chen H.H., Hennessy B.T., Mills G.B. (2012). Mechanistic basis for overcoming platinum resistance using copper chelating agents. Mol. Cancer Ther..

[113] Brem S., Grossman S.A., Carson K.A., New P., Phuphanich S., Alavi J.B., Mikkelsen T., Fisher J.D., New Approaches to Brain Tumor Therapy CNS Consortium (2005). Phase 2 trial of copper depletion and penicillamine as antiangiogenesis therapy of glioblastoma. Neuro-Oncology.

[114] Brady D.C., Crowe M.S., Greenberg D.N., Counter C.M. (2017). Copper Chelation Inhibits BRAF(V600E)-Driven Melanomagenesis and Counters Resistance to BRAF(V600E) and MEK1/2 Inhibitors. Cancer Res..

[115] Garber K., BIOMEDICINE (2015). Targeting copper to treat breast cancer. Science.

[116] Fu S., Hou M.M., Wheler J., Hong D., Naing A., Tsimberidou A., Janku F., Zinner R., Piha-Paul S., Falchook G. (2014). Exploratory study of carboplatin plus the copper-lowering agent trientine in patients with advanced malignancies. Investig. New Drugs.

[117] Fu S., Naing A., Fu C., Kuo M.T., Kurzrock R. (2012). Overcoming platinum resistance through the use of a copper-lowering agent. Mol. Cancer Ther..

[118] Siddik Z.H. (2003). Cisplatin: Mode of cytotoxic action and molecular basis of resistance. Oncogene.

[119] Cossa G., Gatti L., Zunino F., Perego P. (2009). Strategies to improve the efficacy of platinum compounds. Curr. Med. Chem..

[120] Kuo M.T., Chen H.H., Song I.S., Savaraj N., Ishikawa T. (2007). The roles of copper transporters in cisplatin resistance. Cancer Metastasis Rev..

[121] Klug A. (2010). The discovery of zinc fingers and their applications in gene regulation and genome manipulation. Annu. Rev. Biochem..

[122] Kelland L. (2007). The resurgence of platinum-based cancer chemotherapy. Nat. Rev. Cancer.

[123] Jayson G.C., Kohn E.C., Kitchener H.C., Ledermann J.A. (2014). Ovarian cancer. Lancet.

[124] Chen S.J., Kuo C.C., Pan H.Y., Tsou T.C., Yeh S.C., Chang J.Y. (2016). Desferal regulates *hCtr1* and transferrin receptor expression through Sp1 and exhibits synergistic cytotoxicity with platinum drugs in oxaliplatin-resistant human cervical cancer cells in vitro and in vivo. Oncotarget.

[125] Brewer G.J. (2014). The promise of copper lowering therapy with tetrathiomolybdate in the cure of cancer and in the treatment of inflammatory disease. J. Trace Elem. Med. Biol..

